# Effects of Magnetically Guided, SPIO-Labeled, and Neurotrophin-3 Gene-Modified Bone Mesenchymal Stem Cells in a Rat Model of Spinal Cord Injury

**DOI:** 10.1155/2016/2018474

**Published:** 2015-11-16

**Authors:** Rui-Ping Zhang, Ling-Jie Wang, Sheng He, Jun Xie, Jian-Ding Li

**Affiliations:** ^1^Department of Radiology, First Hospital of Shanxi Medical University, Taiyuan 030001, China; ^2^Department of Molecular Biology, Shanxi Medical University, Taiyuan, Shanxi 030001, China

## Abstract

Despite advances in our understanding of spinal cord injury (SCI) mechanisms, there are still no effective treatment approaches to restore functionality. Although many studies have demonstrated that transplanting *NT3* gene-transfected bone marrow-derived mesenchymal stem cells (BMSCs) is an effective approach to treat SCI, the approach is often low efficient in the delivery of engrafted BMSCs to the site of injury. In this study, we investigated the therapeutic effects of magnetic targeting of *NT3* gene-transfected BMSCs via lumbar puncture in a rat model of SCI. With the aid of a magnetic targeting cells delivery system, we can not only deliver the engrafted BMSCs to the site of injury more efficiently, but also perform cells imaging in vivo using MR. In addition, we also found that this composite strategy could significantly improve functional recovery and nerve regeneration compared to transplanting *NT3* gene-transfected BMSCs without magnetic targeting system. Our results suggest that this composite strategy could be promising for clinical applications.

## 1. Introduction

On a worldwide scale, a total of 2.5 million people have suffered spinal cord injury (SCI), which has placed a significant burden on societal resources [1]. Over the past two decades, the understanding of the pathophysiology of SCI has been greatly improved, and significant advances have been made in approaches employing various cell types (e.g., embryonic stem cells, mesenchymal stem cells, and neural stem cells) for transplantation to treat SCI [2]. Many studies have focused on bone marrow mesenchymal stem cell (BMSC) transplantation for SCI because of the pluripotent differentiation capabilities of these cells [3] and because they are associated with fewer ethical issues. Engrafted BMSCs can promote nerve fiber regeneration and functional recovery in animal studies [4]. In addition, neurotrophins play a significant role in modulating neuronal survival, neurogenesis, and synapse formation following SCI [5]. These neurotrophins include brain-derived neurotrophic factor (BDNF), nerve growth factor (NGF), neurotrophin-3 (*NT3*), neurotrophin-4/5 (*NT4/5*), and neurotrophin-6 (*NT6*), many of which can be secreted by BMSCs.

However, BMSC transplantation for SCI cannot always achieve the desired outcome, partly because the number of surviving transplanted cells in injured spinal cord lesions and the secreted neurotrophins can directly influence the effect of cell transplantation [6]. Therefore, we designed an approach that is able to not only improve the efficiency of the delivery of BMSCs to the site of injury but also promote the secretion of neurotrophins.

Magnetic targeting systems with magnetically labeled cells have been evaluated as a more efficient and effective method for the delivery of cells to target sites [6–10]. A previous study by our group demonstrated that transplantation of* NT3*-expressing BMSCs has beneficial effects on functional recovery and nerve regeneration after SCI in a rat model [11]. Therefore, we attempted to combine the two strategies for the treatment of SCI.

The present study was designed to evaluate the therapeutic effects of a magnetic targeting system guiding magnetically labeled BMSCs, combined with* NT3* gene overexpression, as a novel approach for the treatment of SCI.

## 2. Materials and Methods

This study was approved by the Institutional Animal Use and Care Committee of Shanxi Medical University. Eight-week-old female Sprague-Dawley rats (Animal Center of Chinese People's Army Military Medical and Scientific Academy, Beijing, China, SCXK2007-004) were used in our study.

### 2.1. BMSC Isolation and Culture

Donor rats were euthanized with an overdose of pentobarbital sodium administered via intraperitoneal injection. The tibiae and femurs, including both ends of the bones, were harvested. After the proximal and distal ends of the bones were removed, the bone marrow cavities were exposed and flushed repeatedly with 6 mL of Dulbecco's modified essential medium (DMEM; Hyclone, Logan, UT, USA) using a 25-gauge needle. The collected bone marrow suspension was seeded into 25 cm^2^ culture flasks in DMEM containing 10% fetal bovine serum (Hyclone), penicillin (100 U/mL; Invitrogen, Carlsbad, CA, USA), and streptomycin (100 *μ*g/mL, Invitrogen), and the suspension was cultured at 37°C in a 5% CO_2_ incubator (NAPCO, Thermo Scientific, Waltham, MA, USA). After 48 h, nonadherent cells and tissue fragments were discarded by replacing the medium. The medium was subsequently changed at intervals of 2 or 3 days. At approximately 2 weeks after the initiation of seeding, the adherent cells usually reached confluence. Then, the cells were resuspended in a trypsin/EDTA solution (Hyclone, USA) and passaged 1 : 3 at a density of 6,000/cm^2^. After the cells were passaged three times, BMSCs from passage 3 were used for the present study. Details of identification of BMSCs are provided as supplementary data (in Supplementary Material available online at http://dx.doi.org/10.1155/2016/2018474).

### 2.2. Lentiviral Infection of BMSCs

The rat* NT3* gene (reference sequence: NM_031073.2) was synthesized by Nanjing GenScript Bioengineering Technology and Services Co., Ltd. (Nanjing, China), and was confirmed via sequencing. The target plasmids pLV.EX3d.P/puro-EF1*α* >* NT3* > IRES/DsRed Express2 and pLV.EX2d.P/puro-EF1*α* > DsRed Express2 were constructed by Cyagen Biosciences Inc. (Suzhou, China). The target plasmids, three helper plasmids (pLV/helper-SL3, pLV/helper-SL4, and pLV/helper-SL5) and Lipofectamine 2000 were used to create NTF3-DsRed lentivirus and DsRed lentivirus in 293T cells. The titers of the* NT3*-DsRed lentivirus and the DsRed lentivirus ranged from 1 to 1.8 × 10^8^ TU/mL and 3.5 to 3.8 × 10^8^ TU/mL, respectively, and were determined by the plate counting test dilution method.

The packaged lentiviruses were added to passage 3 BMSCs with complete culture medium and then incubated at 37°C in a 5% CO_2_ and 95% relative humidity incubator (NAPCO, Thermo Scientific, Waltham, MA, USA). After 16 h, the medium was replaced. After 48 h, the BMSCs containing the* NT3-DsRed* or* DsRed* gene were harvested, and the* NT3-DsRed* and* DsRed* genes were detected using a fluorescence microscope (IX71, Olympus, Tokyo, Japan). The* NT3* gene was detected via real-time Q-PCR (AB7500, Applied Biosystems, USA).

### 2.3. BMSC Labeling with Superparamagnetic Iron Oxide (SPIO)

In total, 25 *μ*g Fe/mL SPIO (0.5 mmol/mL; Resovist, Schering, Germany) and 375 ng/mL poly-L-lysine (PLL; Sigma, USA) were added to the complete culture medium and incubated at room temperature for 60 min. Then, this medium was added to transfected BMSCs containing the* NT3-DsRed* gene. After the cells were cultured for 24 h at 37°C in a 5% CO_2_ incubator, this medium was discarded. The BMSCs were subsequently washed 3 times with phosphate-buffered saline (PBS) to remove the unlabeled SPIO. Following trypsinization, the BMSCs were suspended in culture medium as the injection solution for transplantation. Details of induction of SPIO-labeled BMSCs are provided as supplementary data.

### 2.4. SCI Model Preparation

We used 8–10-week-old adult female Sprague-Dawley rats (weight 180−240 g) for the present in vivo study. After the rats were anesthetized via intraperitoneal injection of pentobarbital sodium (50 mg/kg), laminectomies were performed microscopically on the T7-8 vertebrae through the midline, with the dura mater intact. A weight-drop device was used to generate the SCI model [12]. To induce a contusion of the spinal cord, a 25 g metal rod with a 2 mm diameter was dropped from a height of 3 cm vertically onto the exposed spinal cord, after which the metal rod was immediately removed from the impact point. Simultaneously, the hind limbs of the rats relaxed completely after twitching, indicating that the SCI model had been successfully generated. Then, the incision was sutured tightly in layers. To prevent postoperative infection, the rats were given 2 × 10^5^ U of penicillin via intramuscular injection every day after surgery. In addition, manual emptying of the bladders of the rats after surgery was performed twice a day by squeezing the lower abdomen until recovery of the micturition reflex was observed. Rats without postoperative hind-limb paralysis were excluded from the present study.

### 2.5. Rat Group Allocation and Cell Transplantation via Lumbar Puncture (LP)

One-week after operation, 36 rats were selected as recipients and were randomly assigned to the following 3 groups: (1) BMSC group (BMSC, *n* = 12); (2)* NT3*-BMSC group (*NT3*, *n* = 12); and (3) Magnet and* NT3*-BMSC group (M-*NT3*, *n* = 12). After the rats were anesthetized again, BMSCs were transplanted via lumbar puncture (LP) as described previously [13]. Briefly, the L5 spinous process and the L4-5 ligamentum flavum were partially resected, and the dura was exposed. Then, at the L4-L5 intervertebral space, a microsyringe was advanced into the subarachnoid space, and 40 *μ*L of culture medium containing 1 × 10^6^ cells was injected into the CSF. In the* NT3* and M-*NT3* groups, the rats received SPIO-labeled* NT3*-BMSCs. In the BMSC group, the rats received unlabeled BMSCs. After injection, each rat was kept in the head-down position at a 30° slope for approximately 30 min [8]. Additionally, a slab of neodymium magnet (0.57 T, length 10 mm, width 8 mm, and height 2 mm) was attached to the spine of the rats at the T7 level with medical adhesive tape in the M-*NT3* group, whereas nothing was placed on the back of the rats in the other two groups. After 24 h, the magnets were removed.

### 2.6. Magnetic Resonance Imaging

After the magnets were removed, the rats were anesthetized via intraperitoneal injection of pentobarbital sodium (50 mg/kg), and MR imaging of the spinal cord was performed using a 1.5 T clinical MR imager (GE Medical System, Signa Infinity Twin Speed & Excite Technology, USA) with a circular surface coil (diameter 11 cm). Axial images were obtained using a standard T2-weighted turbo spin-echo sequence. The imaging sequence parameters were as follows: field of view (FOV), 80 × 80 mm^2^; slice thickness, 2 mm; spacing, 0.5 mm; base resolution matrix, 256 × 256; repetition time (TR), 2,000 ms; and effective echo time (TE), 70 ms. To quantify the signal intensity (SI) in the MR images of the injured spinal cord, signal to noise ratios (SNRs) were calculated as SNR = *S* − *S*_*b*_/SD, where *S* represents the SI of the region of interest (ROI); *S*_*b*_ represents the mean SI of the background; and SD represents the standard deviation of the ROI [14].

### 2.7. Evaluation of Hind-Limb Motor Function

After transplantation, the hind-limb motor function of all of the rats was assessed using the Basso, Beattie, and Bresnahan (BBB) locomotor rating scale [15]. The averaged BBB scores were recorded independently on days 1–7 and then every week up to the fifth week by two examiners who were not aware of the group allocation information.

### 2.8. Tissue Harvesting and Preparation for Histological Assessment

On day 35 after cell transplantation, the rats were perfused intracardially with 100 mL of normal saline (NS), followed by 4% paraformaldehyde in a state of deep anesthesia, through intraperitoneal injection of pentobarbital sodium (100 mg/kg). After the spinal cord tissues were dissected, they were placed in 4% paraformaldehyde overnight for postfixation and were then transferred successfully to 10% and 20% sucrose solutions overnight. Each of the spinal cord tissues at the lesion site was resected as a 10 mm long block. The obtained tissue blocks were frozen, and 20 *μ*m and 5 *μ*m thick longitudinal sections were then cut using a cryostat (CM3050, Leica, Wetzlar, Germany). Next, the sections were mounted on glass slides for histological assessment. For western blot analysis, the rats were not perfused, and the spinal cords tissues were freshly harvested.

### 2.9. Hematoxylin-Eosin (HE) Staining for Cystic Cavity Measurements

Five-micrometer-thick longitudinal sections were stained with HE for measurements of the cystic cavity. After the slices were fixed in cold acetone for 30 min, they were sequentially placed in xylene, ethanol, and distilled water. The slices were stained with hematoxylin for 5 min, rinsed with running water, and then placed in HCL-ethanol for 30 s. After the slices were soaked in running water for 15 min, they were counterstained in eosin and rinsed. Finally, the slices were dehydrated in an ascending series of ethanol, cleared in xylene, air-dried, and enveloped with balata (Sinopharm, Shanghai, China). The area of the cystic cavity was measured using a phase-contrast microscope (E100, Nikon, Tokyo, Japan).

### 2.10. Immunofluorescence Staining

Five-micrometer-thick longitudinal frozen sections were used for immunofluorescence staining. After the slices were fixed in cold acetone for 30 min, they were immersed in 0.3% Triton X-100 for 30 min at room temperature and then treated with 10% goat serum for 1 h at room temperature. Next, the slices were incubated overnight at 4°C with a primary antibody against either neurofilament-200 (NF200) or glial fibrillary acidic protein (GFAP) and then incubated for 1 h at room temperature with a secondary goat anti-mouse antibody (DyLight 488 AffiniPure, Earthox Biotechnology, San Francisco, CA, USA). After the slices were rinsed, they were enveloped with DAPI, and 10 min later, the slices were observed under a fluorescence microscope (IX70, Olympus).

### 2.11. Prussian Blue Staining

To identify iron particles in the SPIO-labeled cells, Prussian blue staining was performed on adjacent sections of the same spinal cord tissues. After the slices were fixed in cold acetone for 30 min, they were incubated in Perl's solution for 30 min at 37°C. Next, the slices were cooled, washed three times with PBS, and then counterstained with nuclear fast red. After the slices were washed and air-dried, they were enveloped with balata.

### 2.12. Statistical Analysis

All results are presented as the means ± standard deviation (SD). The BBB scores were analyzed through repeated measures analysis of variance (ANOVA). The data obtained from Prussian blue staining were analyzed with the Mann-Whitney *U* test. All other data were analyzed using one-way ANOVA to identify significant differences among the three groups. Statistical significance was inferred when the *p* value was less than 0.05. All statistical analyses were performed using the SAS 6.12 software program (SAS Institute, Cary, NC, USA).

## 3. Results

### 3.1. In Vitro Expression of DsRed and* NT3* in BMSCs

**Figure 1:**
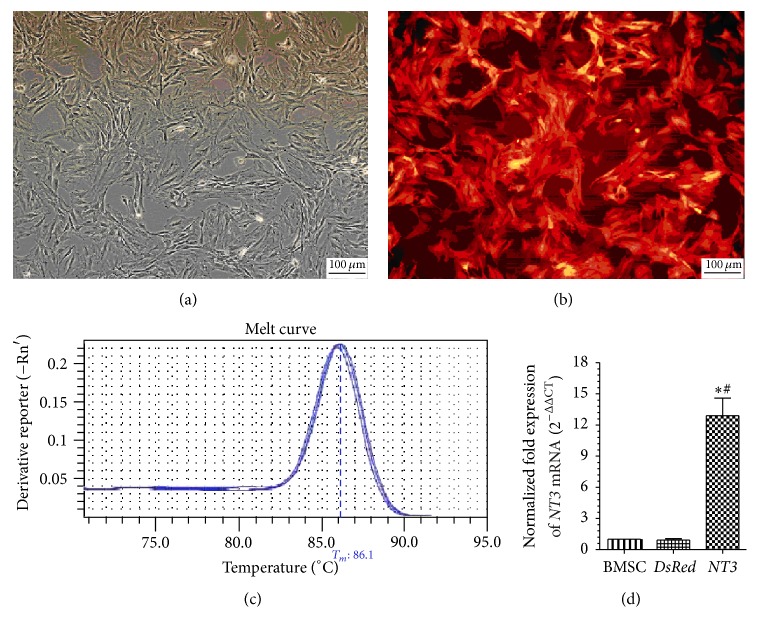
Stable transfection of the* NT3-DsRed* gene into BMSCs using a lentivirus ((a), (b)) and Q-PCT detection of* NT3* mRNA ((c), (d)). (a) Before transfection. (b) After transfection. (c)* NT3* mRNA Q-PCR melting curve. (d) The normalized fold change in* NT3* mRNA expression (2^−ΔΔCT^). Magnification, ×100 ((a), (b)). Scale bar, 100 *μ*m ((a), (b)).

After puromycin screening, red fluorescence was identified in the cytoplasm of >95% of the* NT3*-DsRed BMSCs and >99% of the DsRed BMSCs (Figure 1), which indicated a high transfection efficiency for BMSCs in the present study.

*NT3* mRNA expression was assessed via real-time Q-PCR (Figure 1). The* NT3* mRNA expression levels in the DsRed BMSCs and untransfected BMSCs were nearly equal. The* NT3* mRNA expression level in the* NT3*-DsRed BMSCs was approximately 13-fold that in the DsRed BMSCs or untransfected BMSCs.

### 3.2. Successful Labeling of BMSCs with SPIO

**Figure 2:**
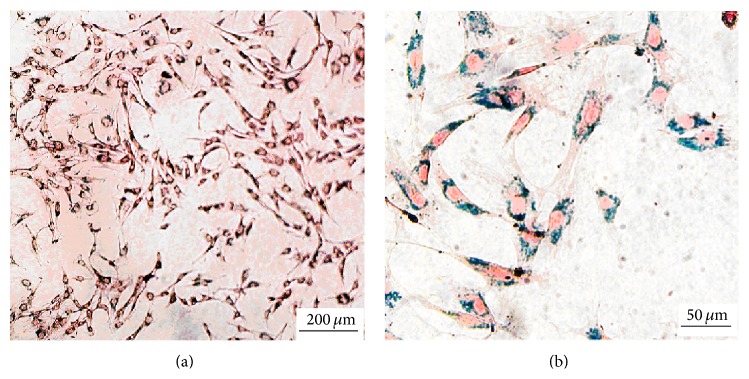
Prussian blue staining of the SPIO-labeled and gene-transfected BMSCs ((a), (b)). Magnification, ×100 (a), ×400 (b). Scale bar, 200 *μ*m (a), 50 *μ*m (b).

After the BMSCs were labeled with SPIO, Prussian blue staining was performed to identify the SPIO labeling rate of BMSCs. Blue iron particles were observed in the cytoplasm of the SPIO-labeled BMSCs, and the SPIO labeling rate of the BMSCs was nearly 100% (Figure 2).

### 3.3. MR Imaging after Cell Transplantation

**Figure 3:**
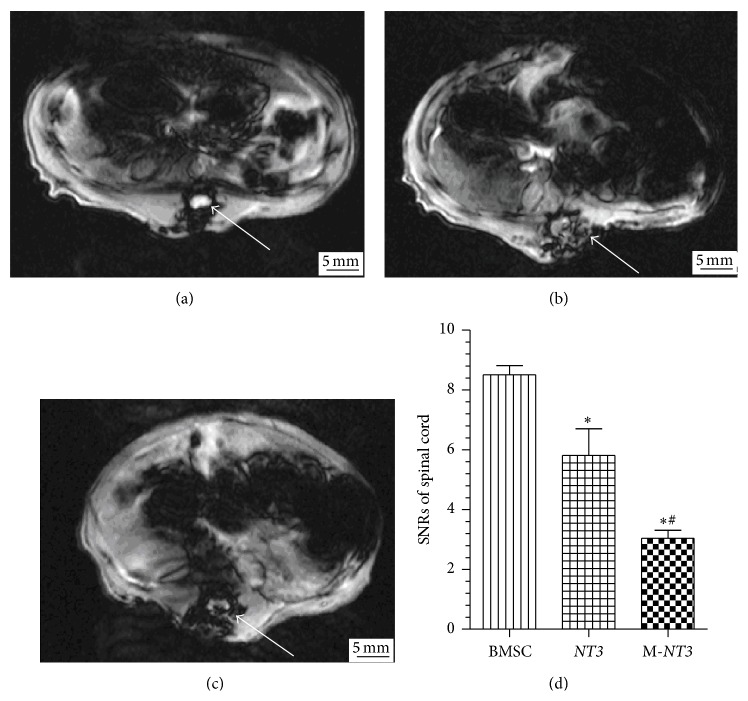
T2^*∗*^-weighted MR images of the injured spinal cord on day 1 after cell transplantation in the BMSC group (a),* NT3* group (b), and M-*NT3* group (c). A bar graph showing the SNRs in the injured spinal cord MR images from each group (d). The data, which are presented as the means ± SD (*n* = 12), were analyzed using one-way ANOVA. ^*∗*^ *p* < 0.05 versus the BMSC group, ^#^ *p* < 0.05 versus the* NT3* group.

The SI was obtained using T2^*∗*^-weighted gradient-echo sequences in the injured spinal cord because T2^*∗*^-weighted imaging is sensitive to SPIO (Figure 3). The injured spinal cord SIs of the M-*NT3* and* NT3* groups decreased after transplantation, whereas no apparent change in the SI was observed in the BMSC group. Analysis of the SNR further revealed that the injured spinal cord SNRs of the M-*NT3* group were significantly decreased compared with those of the* NT3* group, and the SNRs for the injured spinal cords of the* NT3* group were significantly decreased compared with those of the BMSC group.

### 3.4. Locomotor Behavioral Assessment

**Figure 4:**
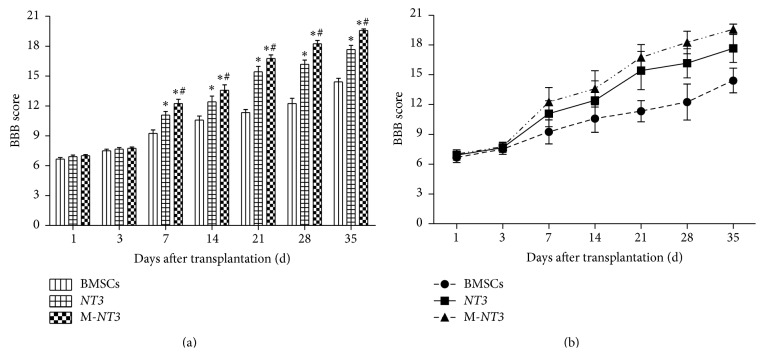
The BBB scores of the hind-limb locomotor functions in each group on days 1, 3, 7, 14, 21, 28, and 35 after cell transplantation ((a), (b)). The data, which are presented as the means ± SD (*n* = 12), were analyzed using repeated measures ANOVA. ^*∗*^ *p* < 0.05 versus the BMSC group, ^#^ *p* < 0.05 versus the* NT3* group.

The hind-limb locomotor functions in each group were assessed using the BBB scores on days 1, 3, 7, 14, 21, 28, and 35 after cell transplantation (Figure 4). On days 1 and 3 after cell transplantation, the BBB scores of the M-*NT3* group did not improve significantly compared with those of the other two groups. However, the BBB scores of the M-*NT3* group were the highest among the three groups on days 7, 14, 21, 28, and 35 after cell transplantation, which indicated that hind-limb locomotor functional recovery was enhanced and accelerated in the M-*NT3* group compared with the other two groups. The BBB scores of the* NT3* group were also significantly higher than those of the BMSC group on days 7, 14, 21, 28, and 35 after cell transplantation. Moreover, significant differences (*p* < 0.05) were observed among the three groups at these time points.

### 3.5. Prussian Blue Staining of the Injured Spinal Cord Tissues

**Figure 5:**
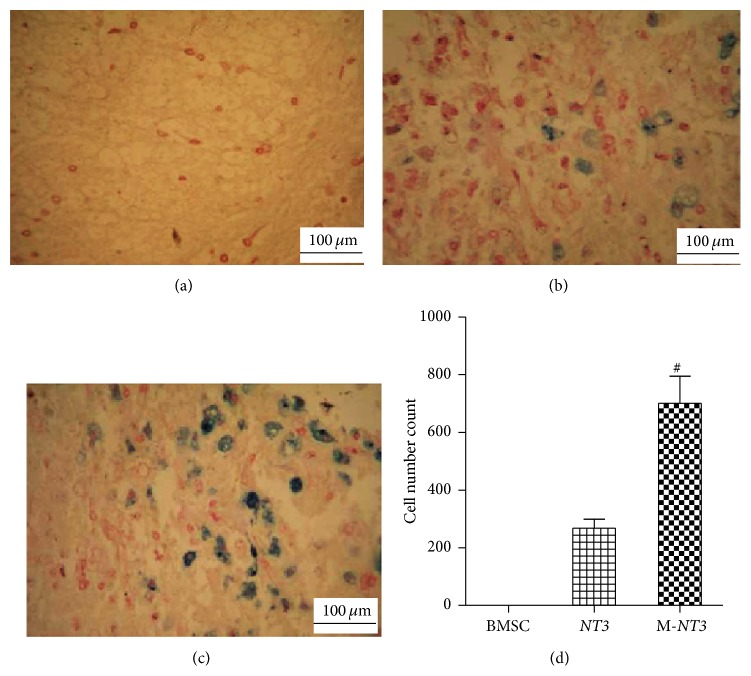
Prussian blue staining of tissues around the injured spinal cord lesion on day 35 after transplantation in the BMSC group (a),* NT3* group (b), and M-*NT3* group (c). Magnification, ×200 ((a), (b), and (c)). Scale bar, 100 *μ*m ((a), (b), and (c)). (d) The data, which are presented as the means ± SD (*n* = 12), were analyzed using the Mann-Whitney *U* test. ^#^ *p* < 0.05 versus the* NT3* group.

After Prussian blue staining was performed on the injured spinal cord tissue sections, significantly more blue-stained cells were observed in the M-*NT3* group than in the* NT3* group, whereas no blue-stained cells could be observed in the BMSC group (Figure 5).

### 3.6. Cystic Cavity Area Measurements

**Figure 6:**
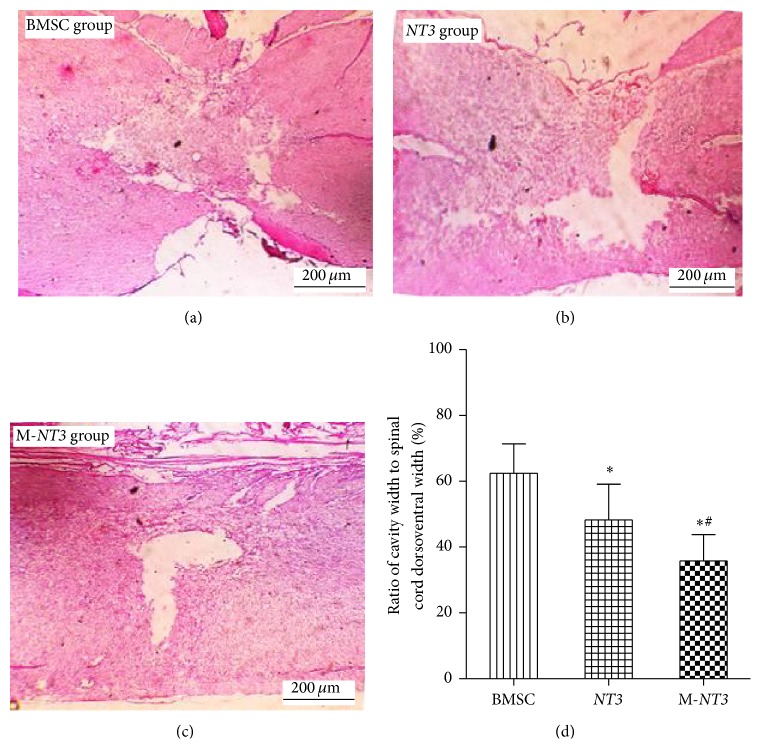
The cystic cavity area of the injured spinal cords on day 35 after cell transplantation in the BMSC group (a),* NT3* group (b), and M-*NT3* group (c). Magnification, ×40 ((a), (b), and (c)). Scale bar, 200 *μ*m ((a), (b), and (c)). The data, which are presented as the means ± SD (*n* = 12), were analyzed using one-way ANOVA. ^*∗*^ *p* < 0.05 versus the BMSC group, ^#^ *p* < 0.05 versus the* NT3* group.

At 7 weeks after the spinal cord was injured, the mean values of the cystic cavity area in the BMSC,* NT3*, and M-*NT3* groups were 0.64 ± 0.14 mm^2^, 0.51 ± 0.11 mm^2^, and 0.39 ± 0.10 mm^2^, respectively. Statistically significant differences were observed among the three groups (*p* < 0.05, Figure 6), with the cystic cavity area in the M-*NT3* group being the smallest.

### 3.7. Expression of the* NT3* Protein in the Injured Spinal Cord after Cell Transplantation

**Figure 7:**
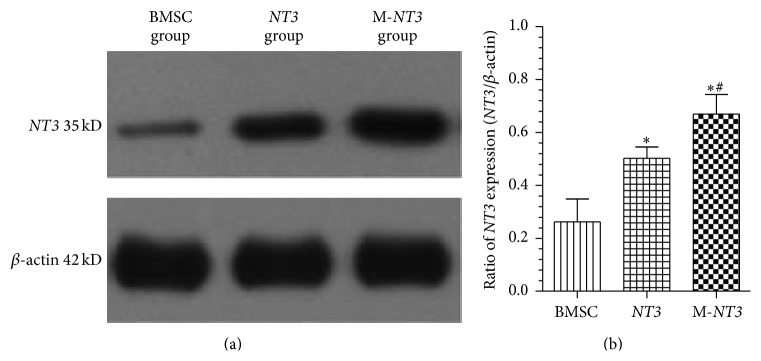
Western blot detection of* NT3* protein expression in the injured spinal cords on day 35 after cell transplantation in each group (a). Ratio of the lane density of the* NT3* protein to the lane density of the *β*-actin protein in each group (b). The data, which are presented as the means ± SD (*n* = 12), were analyzed using one-way ANOVA. ^*∗*^ *p* < 0.05 versus the BMSC group, ^#^ *p* < 0.05 versus the* NT3* group.

On day 35 after cell transplantation,* NT3* protein expression was investigated through western blot analysis (Figure 7). Although* NT3* protein overexpression was observed in both the M-*NT3* group and the* NT3* group, the* NT3* protein level in the M-*NT3* group was significantly higher than in the* NT3* group. In addition, the* NT3* protein level in the BMSC group was significantly lower than in the* NT3* group. The magnetic targeting system significantly enhanced* NT3* protein expression in the injured spinal cord lesions.

### 3.8. Axon Regeneration and Glial Scar Inhibition in the Injured Spinal Cord

**Figure 8:**
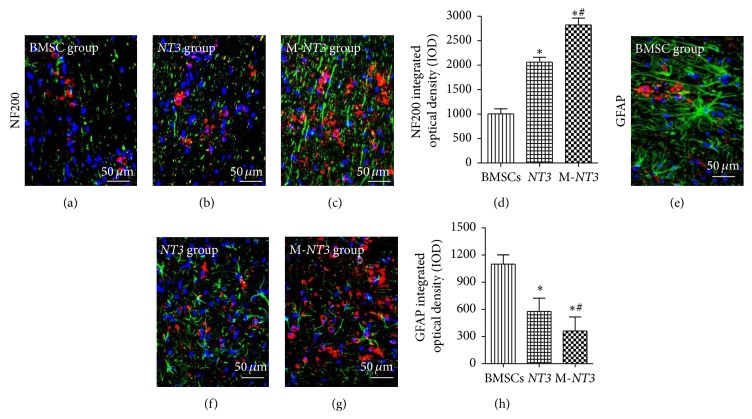
Immunofluorescence labeling of NF200 ((a), (b), and (c)) and GFAP ((e), (f), and (g)) in sagittal sections of the injured spinal cords from each group on day 35 after cell transplantation. Double labeling for NF200/GFAP (green) and BMSCs (red). Magnification, ×400 ((a)–(c), (e)–(g)). Scale bar, 50 *μ*m ((a)–(c), (e)–(g)). Integrated optical density (IOD) bar graphs showing NF200 (g) and GFAP (h) expression in each group. All microscopic images were captured under identical conditions. The data, which are presented as the means ± SD (*n* = 12), were analyzed using one-way ANOVA. ^*∗*^ *p* < 0.05 versus the BMSC group, ^#^ *p* < 0.05 versus the* NT3* group.

NF200 is a NF protein that is found in axons under normal conditions [16]. Increased expression of NF200 indicates axon regeneration in the injured spinal cord. In contrast, GFAP is expressed primarily in astrocytes, and a reduction of its expression indicates glial scar inhibition in the injured spinal cord. As shown in Figure 8, the level of NF200 expression in the M-*NT* group was significantly increased compared with that in the other two groups, whereas the GFAP expression level in the M-*NT* group was significantly decreased compared with that in the other two groups. Moreover, the NF200 expression level in the* NT3* group was higher than in the BMSC group, whereas the GFAP expression level in the* NT3* group was lower than in the BMSC group.

## 4. Discussion

In the present study, we investigated the effects of a magnet-guided SPIO-labeled BMSC transplantation method combined with gene therapy for the treatment of spinal cord injury in rats. We found that this approach not only was more effective for repairing SCI but also allowed the engrafted cells to be tracked in vivo.

SCI is a complex pathological process for which effective treatment strategies are currently lacking [17]. Cell replacement therapy is a promising approach for repairing the injured spinal cord. In particular, BMSCs have been extensively studied in animal models of SCI because these cells are easily isolated and cultured, exhibit pluripotent differentiation capabilities, and are associated with fewer ethical issues [2, 3, 8, 18, 19]. Although many experimental studies have demonstrated that BMSC transplantation can promote nerve regeneration and enhance functional recovery in animal models of SCI [20], the desired outcome (full recovery) cannot be obtained. The possible reasons for these poor outcomes include the lack of adequate neurotropic factors and surviving stem cells in injured spinal cord lesions.

Neurotropic factors, such as nerve growth factor (NGF), brain-derived neurotrophic factor (BDNF), neurotrophin-3 (*NT3*), neurotrophin-4/5 (*NT4/5*), and neurotrophin-6 (*NT6*), play crucial roles in nerve regeneration after SCI [21, 22]. Although BMSCs can express many neurotropic factors in vitro [23, 24], engrafted BMSCs do not express* NT3* in vivo [23, 25, 26]. However,* NT3* is an extremely important neuroregenerative protein involved in guiding stem cell migration, mediating engrafted BMSC survival, inducing neuronal differentiation, promoting axonal regeneration, and facilitating angiogenesis after SCI [22, 27–31]. Considering these* NT3* protein activities, stably* NT3-*transfected BMSCs were obtained using lentivirus vectors to produce BMSCs that could overexpress the* NT3* protein in vivo.

The present study showed that stable transfection of BMSCs with the* NT3* gene was possible, and the resulting cells were more effective than conventional BMSCs in vivo for promoting axonal regeneration, enhancing functional recovery, and maintaining the survival of engrafted cells. Although the* NT3* protein could be detected in the BMSC group, it was expressed at a much lower level than in the other two groups. This low-level expression may be attributed to the presence of inflammatory cells (i.e., macrophages, lymphocytes, and mast cells), microglia/macrophages, astrocytes, oligodendrocytes, and neural progenitor cells that are present following spinal cord injury and can express the* NT3* protein [32–36]. All of these findings are consistent with a previous study by our group [11]. In addition, our previous study demonstrated that* NT3* gene-modified BMSC transplantation was superior to BMSC transplantation in promoting neuronal regeneration, inhibiting glial scar formation, and increasing BDNF and VEGF secretion after SCI [11].

The survival of the engrafted stem cells in the lesion has a direct effect on the repair outcome of stem cell transplantation strategies [6, 37]. Although BMSCs have been shown to home to injured spinal cord lesions [38, 39], this homing effect does not result in an adequate number of BMSCs homing to the lesion for clinical efficacy. Direct injection, lumbar subdural injection, and intravessel injection are the traditional methods used for cell transplantation. Many recent studies have shown that lumbar subdural injection is a safe, less invasive (compared to the other methods), and repeatable method of cell transplantation [8, 13, 40, 41]. However, the direct injection of cells is more efficient than lumbar subdural injection [8], although this method is more invasive than intravessel injection.

A highly efficient and less invasive cell delivery system is particularly important for cell transplantation to treat SCI. To achieve a compromise between efficiency and invasiveness, we chose to transplant cells via lumbar subdural injection in the present study. To improve the efficiency of cell delivery, a magnetic targeting system was used. Some previous studies demonstrated that SPIO-labeled BMSCs transplanted via lumbar puncture were delivered to the spinal cord injury site efficiently through the CSF using a magnetic targeting system [6, 8, 10]. In addition, SPIO nanoparticles can significantly shorten T1 and T2 relaxation times and particularly the T2^*∗*^ relaxation time. Therefore, SPIO nanoparticles can cause a significant reduction of the T2^*∗*^ SI observed under MRI. In the present study, many more blue-stained cells could be observed following Prussian blue staining, and the injured spinal cord SNRs of the T2^*∗*^-weighted MR images were significantly decreased in the magnet group compared with the other two nonmagnet groups; both of these findings indicated that more BMSCs homed to the injured spinal cord lesions in the magnet group than in the other two, nonmagnet groups.

NF200 is one of the NF proteins that can be found in axons under normal conditions [15]. After SCI, astrocytosis will increase GFAP expression following glial scar formation in the lesion. The immunofluorescence experiments performed in the present study showed that NF200 expression around the injured site was much higher in the magnet group than in the other two, nonmagnet groups, whereas GFAP expression around the injured site was much lower in the magnet group than in the other two, nonmagnet groups. These findings indicate that transplantation of* NT3* gene-modified BMSCs with the aid of a magnetic targeting system can significantly promote neuronal regeneration and inhibit glial scar formation.

Locomotor testing was also performed in the present study to assess hind-limb motor function using the BBB score. At day 35 after cell transplantation, the BBB scores of the magnet group did not appear to be markedly improved compared with the* NT3*-BMSC group from our previous study [11], which was mainly attributed to the different researchers involved in the two studies and the subjectivity of the BBB scoring system. Although the BBB scores of the magnet group were improved by only approximately 10% at day 35 after cell transplantation in the present study, the dynamic variation of BBB scores in the three groups indicated that hind-limb locomotor functional recovery was enhanced and accelerated in the magnet group compared with the other two, nonmagnet groups. All of these findings could be attributed primarily to the magnetic targeting system effectively delivering the engrafted BMSCs to the injured spinal cord lesion [10].

In summary, the present study demonstrated that the Magnet-*NT3* group showed a significantly better efficiency of cell delivery, nerve regeneration, and functional recovery than the* NT3*-BMSC group. Thus, transplantation of* NT3* gene-modified BMSCs via lumbar puncture combined with a magnetic targeting system is a highly effective and minimally invasive therapeutic method for treating spinal cord injury and is promising for monitoring engrafted BMSCs in vivo through MR.

## Supplementary Material

The cells identification using flow cytometry showed that the surface markers of the cells were positive for CD44 and CD90, but negative for CD34, according with the expression of BMSC surface markers. After induced neural differentiation of SPIO-labeled BMSCs in vitro, the cells presented the appearance of neural cells and expressed NSE (neuron-specific enolase).
