# Supplementary material for: Effects of Magnetically Guided, SPIO-Labeled, and Neurotrophin-3 Gene-Modified Bone Mesenchymal Stem Cells in a Rat Model of Spinal Cord Injury
Source: Stem Cells Int. 2015 Nov 16;2016:2018474. doi: 10.1155/2016/2018474 (PMC4663356; doi:10.1155/2016/2018474)
Supplement: Supplementary file 1 — The cells identification using flow cytometry showed that the surface markers of the cells were positive for CD44 and CD90, but negative for CD34, according with the expression of BMSC surface markers. After induced neural differentiation of SPIO-labeled BMSCs in vitro, the cells presented the appearance of neural cells and expressed NSE (neuron-specific enolase). [file 2018474.f1.doc]

**Supplementary**

**BMSC identification using flow cytometry**

BMSCs were identified using flow cytometry (BD Accuri C6, BD, USA). Briefly, BMSCs from passage 3 were digested using trypsin and were collected, resuspended and diluted to a concentration of 1×10^6^ cells/ml. After 100 μl of a single cell suspension was incubated with FITC-labeled mouse anti-CD44 or anti-CD90 or PE-labeled mouse anti-CD34 (BD Pharmingen, San Diego, CA, USA) for 20 min in the dark at room temperature, the cells were washed with phosphate‑buff­ered saline (PBS) and analyzed via flow cytometry. The outcomes of the flow cytometric analysis showed that the surface markers of the cells were positive for CD44 and CD90, but negative for CD34, which indicated that the cells expressed high levels of mesenchymal stem cell surface markers but were negative for hematopoietic surface markers (Figure 1).

**
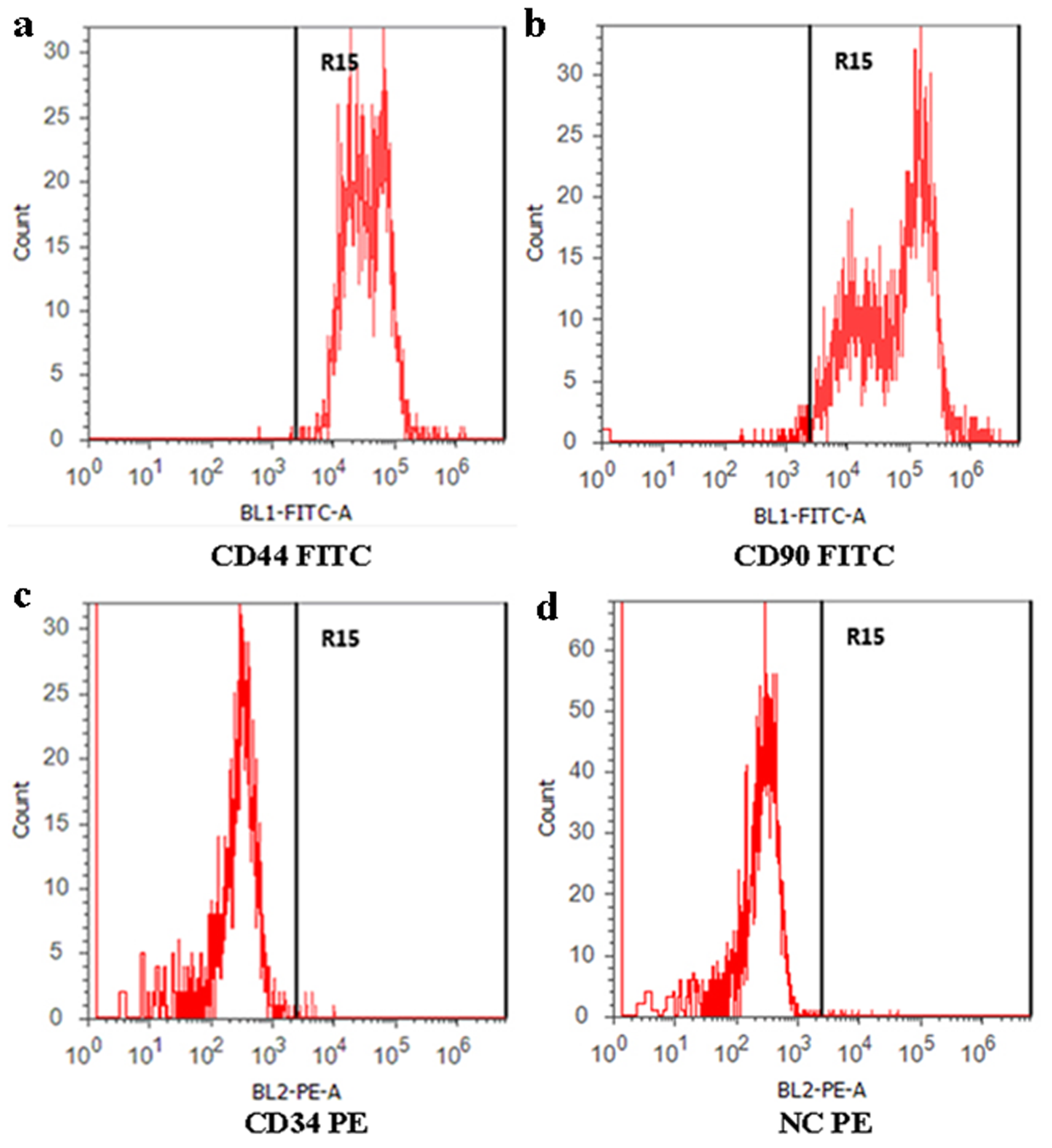
**

**Figure 1.** Flow cytometric analysis of BMSC surface markers. **(a)** CD44 (positive), **(b)** CD90 (positive), **(c)** CD34 (negative), and **(d)** Normal Control (negative).

**In vitro induced neural differentiation of SPIO-labeled** **BMSCs**

SPIO-labeled passage-3 BMSCs were pre-induced with 5 ml of 1 mmol/L mercaptoethanol (Sigma, St. Louis, MO, USA) for 24 hours. Then, the cells were rinsed three times with PBS and cultured with serum-free medium (Hyclone) containing 40 ng/ml basic fibroblast growth factor (bFGF, Sigma) for 7 days. After the differentiation of BMSCs was induced, a neuronal marker (neuron-specific enolase, NSE) was detected via immunocytochemical staining, and iron particles were detected by Prussian blue staining (Figure 2). After differentiation was induced, the BMSCs presented the appearance of neural cells and exhibited intercellular protuberances that were connected with each other. Immunostaining showed that the induced cells were brown, which indicated that the cells expressed NSE. The cytoplasm of the BMSCs was stained blue by Prussian blue staining. Therefore, the SPIO-labeled BMSCs show neural differentiation capabilities.


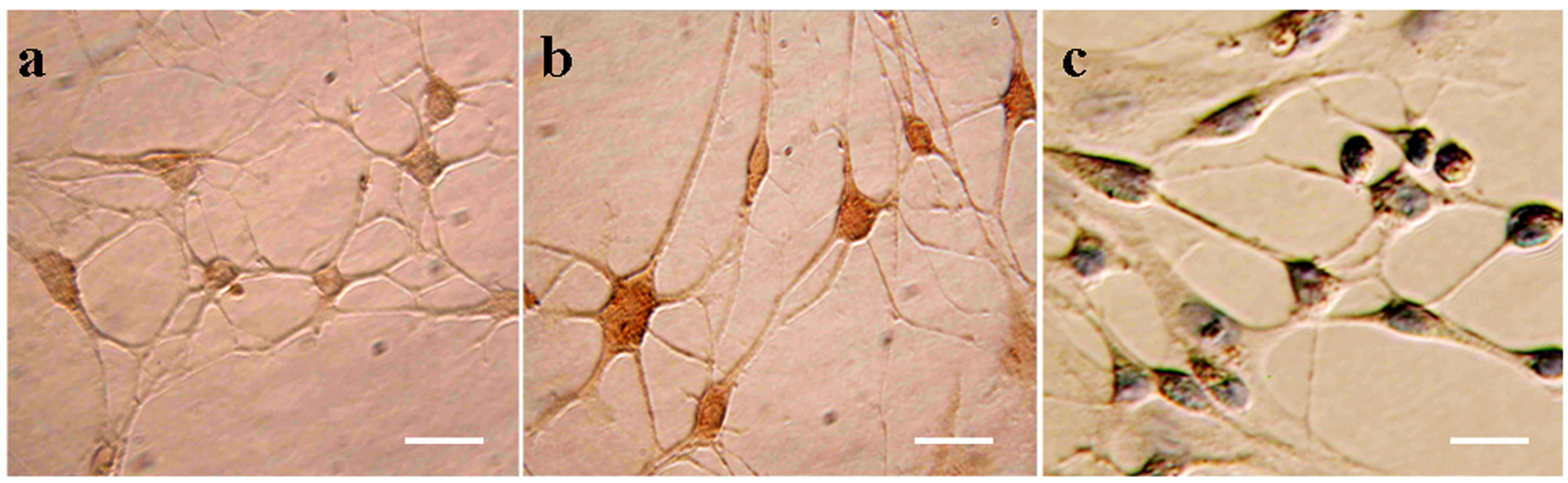


**Figure 2.** Neural differentiation of SPIO-labeled BMSCs. After differentiation was induced, the morphology of BMSCs was similar to that of neural cells with intercellular protuberances **(a)**; the observation of brown BMSCs following immunocytochemical staining indicated that NSE was expressed **(b)**; the blue-stained cytoplasm of the BMSCs indicated the presence of iron particles **(c)**. Magnification, ×400 (**a, b, c)**. Scale bar, 50 μm **(a, b, c)**.
